# Utilizing Selected Di- and Trinucleotides of siRNA to Predict RNAi Activity

**DOI:** 10.1155/2017/5043984

**Published:** 2017-01-24

**Authors:** Ye Han, Yuanning Liu, Hao Zhang, Fei He, Chonghe Shu, Liyan Dong

**Affiliations:** ^1^Department of Computer Science and Technology, Jilin University, Changchun, Jilin, China; ^2^Key Laboratory of Symbolic Computation and Knowledge Engineering, Ministry of Education, Jilin University, Changchun, China; ^3^Department of Computer Science and Information Technology, Northeast Normal University, Changchun, Jilin, China; ^4^Department of Environment, Northeast Normal University, Changchun, Jilin, China; ^5^Institute of Computational Biology, Northeast Normal University, Changchun, China

## Abstract

Small interfering RNAs (siRNAs) induce posttranscriptional gene silencing in various organisms. siRNAs targeted to different positions of the same gene show different effectiveness; hence, predicting siRNA activity is a crucial step. In this paper, we developed and evaluated a powerful tool named “siRNApred” with a new mixed feature set to predict siRNA activity. To improve the prediction accuracy, we proposed 2-3NTs as our new features. A Random Forest siRNA activity prediction model was constructed using the feature set selected by our proposed Binary Search Feature Selection (BSFS) algorithm. Experimental data demonstrated that the binding site of the Argonaute protein correlates with siRNA activity. “siRNApred” is effective for selecting active siRNAs, and the prediction results demonstrate that our method can outperform other current siRNA activity prediction methods in terms of prediction accuracy.

## 1. Introduction

RNA interference (RNAi) is a cellular process whereby double-stranded RNA (dsRNA) leads to posttranscriptional gene silencing through base-pairing interactions and is found in many eukaryotic systems, including plants, fungi, invertebrates, and mammals [1–4]. In mammalian cells, long dsRNA is processed into short 21–23 nucleotide (nt) dsRNAs known as small interfering RNA (siRNA) and induces instant target gene knockdown [3]. In functional genomic research, RNAi has become very helpful in drug and therapeutic applications [5]. Highly effective siRNAs can be synthesized to design novel drugs for influenza virus [6], HIV virus [7], and cancer [8]. However, Takayuki measured the RNAi activities of siRNAs targeting all positions of a single mRNA in human cells and found that few siRNAs show very high activities [9]. Therefore, predicting siRNA activity is a critical step for the successful implementation of RNAi.

Numerous siRNA-designing algorithms, which can be generally categorized as first-and second-generation algorithms, have been reported to date. The first-generation algorithms are based on small validated siRNA datasets and exploit multiple siRNA features, including GC content [10], base preferences at specific positions [11, 12], thermodynamic stability [13], internal structure [14], and target mRNA secondary structure [15–17]. However, a large majority of siRNAs designed by the first-generation algorithms are not very effective [18]. The reason may be that the early datasets are too small to cover all the important features [19].

The second-generation algorithms were developed with the accumulation of validated siRNAs. Huesken developed “Biopredsi” [20] based on artificial neural network and built a major siRNA dataset including 2431 siRNAs through high-throughput analysis technology. A number of siRNA activity prediction algorithms based on machine learning models were built using Huesken's dataset. The algorithms ThermoComposition21 [21], DSIR [22], *i*-score [23], and Biopredsi were estimated as the best predictors [24]. In addition, Takayuki et al. proposed a complete dataset including the siRNAs targeting all positions of a single mRNA in human cells and developed an algorithm “siExplored.” They found that specific residues at every third position of siRNAs greatly influenced its RNAi activity [9].

The performance of second-generation algorithms heavily depends on the selection of the included features [25]. Because the siRNA sequence is the most important factor that determines RNAi activity, more potential features embedded in siRNA sequences should be exploited to increase prediction accuracy. Takahashi found that when the 2-3 bp RNA at every position of a siRNA sequence were substituted by DNA, the RNAi activity changed [26]. Thus, we consider that the di- and trinucleotides at certain positions of siRNA may correlate with its RNAi activity.

In this paper, we developed a powerful siRNA activity predictor by fusing multiple potential features. Our experimental results demonstrate that siRNA activity is significantly affected by its di- and trinucleotides; thus, we proposed 2-3NTs as our new features. In addition, a new mixed 230-dimensional feature set was formed by combining 191 traditional features and 39 new features. To select the most relevant features, we proposed a Binary Search Feature Selection (BSFS) algorithm. Finally, a Random Forest predictor is constructed using the selected features. At the same time, a user-friendly web server named siRNApred is developed and is available for free at http://www.jlucomputer.com:8080/RNA/. siRNApred showed better performance compared with first-generation and second-generation algorithms. The result suggests that the di- and trinucleotides of siRNA can provide important information for prediction of active siRNAs.

## 2. Materials and Methods

### 2.1. Dataset

Huesken's dataset includes [20] 2431 siRNAs targeted to 34 human and rodent mRNAs. The dataset is divided into the 2182-sequence training set (Huesken_train) and 249-sequence testing set (Huesken_test). Three independent datasets from Vickers, Reynolds, and Haborth, including 368 siRNAs, are used for testing [11, 27, 28].

### 2.2. The Importance of the Di- and Trinucleotides of siRNA

In this section, we first elucidated the importance of our proposed di- and trinucleotides of siRNA on its activity. The di- and trinucleotides of siRNA can be defined as follows:

The guide strand of siRNA *S* = *a*_1_, *a*_2_,…, *a*_*i*_,…, *a*_21_, where 1 ≤ *i* ≤ 21.
*a*_*d*_*a*_*d*+1_ represents the dinucleotide at position *d*, where 1 ≤ *d* ≤ 20.
*a*_*t*_*a*_*t*+1_*a*_*t*+2_ represents the trinucleotide at position *t*, where 1 ≤ *t* ≤ 19.

All di- and trinucleotides at all positions of siRNA are obtained by a sliding window size of 2-3. Huesken's dataset is divided into two classes: 1218 potent siRNAs with activities greater than 0.7 and 1213 nonpotent siRNAs with activities less than 0.7.

There are 16 2-mer RNA subsequences, that is, AA, AU, etc., and the frequencies of all 2-mer RNA subsequences at positions 1 to 20 are calculated for the two classes. The significance level is calculated by Student's *t*-test and the 2-mer RNA subsequences with minimal *p* value are shown in Table 1 (*p*-value < 0.05).

Table 1 shows that the 2-mer RNA subsequences that appeared most often as potent were different than those that appeared most often as nonpotent siRNAs. We found that “UU” occurred more often than other 2-mer RNA subsequences in potent siRNAs, whereas “GG” and “CC” appeared most often in nonpotent siRNAs. Most of the “UU” 2-mers were found at positions 1, 4, 6, and 7 of potent siRNAs. In nonpotent siRNAs, “GG” often occurred at positions 1, 13, 14, 15, and 16 and “CC” often occurred at positions 3, 4, 5, 6, and 20.

There are 64 3-mer RNA subsequences, that is, AAA, AAU, etc. In addition, the frequencies of all 3-mer RNA subsequences at positions 1 to 19 are calculated for the two classes. The significance level is calculated by Student's *t*-test and the 3-mer RNA subsequences with minimal *p* value are shown in Table 2 (*p* value < 0.05).

The results demonstrate that di- and trinucleotides of siRNAs at certain positions can be used as indicators to distinguish between potent siRNAs and nonpotent siRNAs and can possibly be used as a potential feature for siRNA activity prediction.

### 2.3. Feature Extraction

A total of 230 features are extracted in this section for siRNA activity prediction. These features include 2-3NTs, thermodynamic stability, nucleotide representation, and nucleotide compositions.

#### 2.3.1. 2-3NTs

2-3NTs are categorical features extracted from the di- and trinucleotides of siRNAs.

We defined the feature vector *X*_2NT_ including 20 categorical features extracted from the dinucleotides of siRNA as follows:

(1) X2NT=Ca1a2,…,Capositionaposition+1,…,Ca20a21,

where 1 ≤ position ≤ 20.

The categorical feature *C*(*a*_position_*a*_position+1_) is calculated using the following formula:

(2) $$\begin{array}{c} C\left(\;a_{position}a_{position+1}\;\right)=\left(\;f-1\;\right)\times 4+s, \end{array}$$

where

(3) f=1if  aposition= “A” 2if  aposition=“U”or  aposition=“T”3if  aposition=“G”4if  aposition=“C”,s=1if  aposition+1=“A”2if  aposition+1=“U”or  aposition+1=“T”3if  aposition+1=“G”4if  aposition+1=“C”.

Then, the feature vector *X*_3NT_, which includes 19 categorical features, is extracted from the trinucleotides of siRNA as follows:

(4) X3NT=Ca1a2a3,…,Capositionaposition+1aposition+2,…,Ca19a20a21,

where 1 ≤ position ≤ 19.

The categorical feature *C*(*a*_position_*a*_position+1_*a*_position+2_) is calculated using the following formula:

(5) Capositionaposition+1aposition+2=f−1×16+s−1×4+t,

where

(6) f=1if  aposition+1=“A”2if  aposition+1=“U”or  aposition+1=“T”3if  aposition+1=“G”4if  aposition+1=“C”,s=1if  aposition+1=“A”2if  aposition+1=“U”or  aposition+1=“T”3if  aposition+1=“G”4if  aposition+1=“C”,t=1if  aposition+1=“A”2if  aposition+1=“U”or  aposition+1=“T”3if  aposition+1=“G”4if  aposition+1=“C”.

#### 2.3.2. Thermodynamic Stability

The thermodynamic stability of siRNA may influence the strand selection in the process of RNAi; thus it would influence the RNAi activity [23]. Δ*G*_duplex_ is the sum of all the siRNA local duplex stability. The siRNA local duplex stability is calculated for every two base pairs along the siRNA duplex and the thermodynamic parameters for calculations were supplied by Xia et al. [29]. The ΔΔ*G* is the Δ*G* difference of duplex formation at the 5′ and 3′ ends of siRNA for 5 terminal nucleotides.

#### 2.3.3. Nucleotide Representation

Preferred nucleotides at specific positions are important indicators for activity prediction [21]. For example, the nucleotides at the first position of potent siRNAs were most often *A* or *U*, while *C* often appeared at positions 7 and 11 in nonpotent siRNAs [11, 20]. We defined the siRNA as a 21-dimensional vector and indicated the nucleotides at all positions. *A*, *U*, *G*, and *C* were digitized as 0.1, 0.2, 0.3, and 0.4.

#### 2.3.4. Nucleotide Compositions

The compositions of short motifs of 1–3 nt in siRNA and mRNA contained relevant information for activity prediction [30, 31]. There are 4, 16, and 64 possible subsequences for all 1-mer, 2-mer, and 3-mer RNAs, respectively. Thus, there are 168 features extracted from nucleotide compositions.

### 2.4. Model Construction

Random Forest (RF) [32] is an ensemble learning method for classification and regression by growing a collection of trees. In the process of regression, the trees are constructed using a training set with *M* variables. *m* variables from these *M* input variables are selected for the construction of an individual tree. The mean prediction of the individual tree will be output when the testing samples are pushed down these trees. Because the RF algorithm can randomly select features to build the ensemble of trees, it has stronger robustness than other methods. In this paper, the RF algorithm was used to develop siRNA activity prediction model.

### 2.5. Feature Selection

We combined 39 2-3NTs, 2 thermodynamic stabilities, 21 nucleotide representations, and 168 nucleotide compositions to obtain a 230-dimensional feature vector. Since the contributions of these features are different, we proposed BSFS algorithm based on RF-variable importance to select the optimal feature set. The process of the algorithm is shown as follows.

Firstly, all features are ranked in descending order according to its *z*-score. The *z*-score is calculated by the RF algorithm to measure the feature importance [32]. To get the *z*-score, Variable Importance (VI) should be first calculated.

VI of the *j*th variable was calculated according to the mean decrease in classification accuracy after permuting values of variable *x*_*j*_ over all trees. The VI(*x*_*j*_) of each tree *t* is computed as follows:

(7) VItxj=∑i∈β¯tIyi=y^itβ−t−∑i∈β¯tIyi=y^i,πjtβ−t,

where ${\overset{-}{\beta }}^{\left(t\right)}$ is OOB samples of tree *t*.

(8) $$\begin{array}{c} {\hat{y}}_{i}^{\left(t\right)}=f^{\left(t\right)}\left(\;x_{i}\;\right), \end{array}$$

where *x*_*i*_ is the variable value and ${\hat{y}}_{i}^{\left(t\right)}$ is predicted class before permutation.

(9) $$\begin{array}{c} {\hat{y}}_{i,\pi_{j}}^{\left(t\right)}=f^{\left(t\right)}\left(\;x_{i,\pi_{j}}\;\right), \end{array}$$

where *x*_*i*,*π*_*j*__ = (*x*_*i*,1_,…, *x*_*i*,*j*−1_, *x*_*π*_*j*_(*i*),*j*_, *x*_*i*,*j*+1_,…, *x*_*i*,*p*_) is the variable value after randomly permuting the *j*th variable and ${\hat{y}}_{i,\pi_{j}}^{\left(t\right)}$ is the predicted class after permutation.

Please note that if *X*_*j*_ is not in the tree *t*, then VI^(*t*)^(*x*_*j*_) = 0.

Over all trees, VI(*x*_*j*_) is defined as follows:

(10) $$\begin{array}{c} VI\left(\;x_{j}\;\right)=\frac{\sum_{t=1}^{n\;tree}{VI}^{\left(t\right)}\left(x_{j}\right)}{n\;tree}, \end{array}$$

where *n* tree is the number of trees in the Random Forest.

Finally, the *z*-score of the *j*th feature is defined as follows:

(11) $$\begin{array}{c} z\text{-}{\text{score}}_{j}=\frac{VI\left(x_{j}\right)}{\hat{\sigma }/\sqrt{n\;tree}}, \end{array}$$

where $\hat{\sigma }$ is the standard deviation of the raw importance.

Secondly, the first *k* features are selected as the optimal features. Set *k* < *m* and the calculation process of threshold *k* is summarized in Algorithm 1.

### 2.6. Model Performance Evaluation

As a validation step, we used the Pearson Correlation Coefficient (PCC) to describe the correlation between experimentally determined and predicted siRNA activity. It may be defined as follows:

(12) $$\begin{array}{c} PCC=\frac{1}{n-1}\overset{n}{\underset{i=1}{\sum }}\left(\;\frac{X_{i}-\overset{-}{X}}{\sigma_{X}}\;\right)\left(\;\frac{Y_{i}-\overset{-}{Y}}{\sigma_{Y}}\;\right), \end{array}$$

where *n* is the sample size and $\overset{-}{X}$ and *σ*_*X*_ are the average value and standard deviation, respectively.

In addition, the Receiver Operating Characteristic (ROC) curve is applied to illustrate the performance of a binary classifier system by plotting sensitivity (*Y* axis) against 1 − specificity (*X* axis) at various threshold settings.

(13) Sensitivity=TPTP+FN,Specificity=TNTN+FP,

where TN is the number of true negatives, FN is the number of false negatives, TP is the number of true positives, and FP is the number of false positives.

The area under the ROC curve (AUC) is a single measurement of the algorithm's overall performance, and AUC of 1 and 0.5 represents perfect classification and random classification, respectively.

## 3. Results and Discussion

### 3.1. Performance of the 2-3NTs Features

To investigate the importance of di- and trinucleotides of siRNA, we learn two RF regression models trained using Huesken_train and tested on Huesken_test. “model 1” is constructed with 2 thermodynamic stabilities, 21 nucleotide representations, and 168 nucleotide compositions, which are often used for siRNA activity prediction [24]. Then, “model 2” which extended “model 1” by considering 39 2-3NTs was constructed for comparisons.

**Figure 1:**
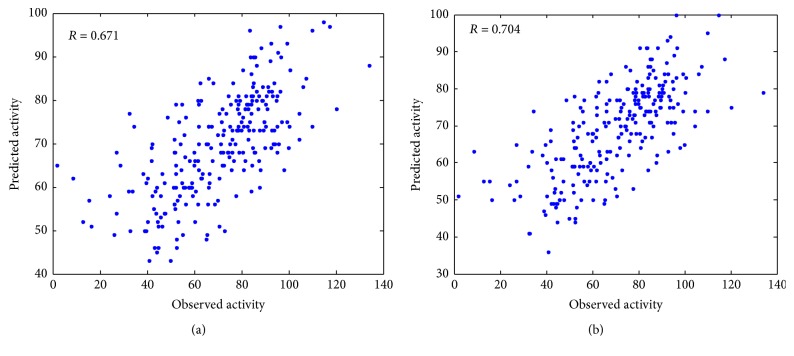
Comparison between model 1 and model 2. Observed siRNA activities of the Huesken_test are plotted against predicted siRNA activities by model 1 (a) and model 2 (b).

The experimental prediction results are shown in Figure 1, and the PCC between the observed and predicted siRNA activities for model 1 and model 2 are 0.671 and 0.704, respectively. The prediction efficacy achieved 4.92% improvement after adding the new proposed features. It validates that 2-3NTs are important features for the prediction of siRNA activity.

### 3.2. Feature Selection Result

The optimal feature set is obtained by our proposed BSFS algorithm. The details of this algorithm are shown in Section 2.5.

Table 3 shows the threshold “*k*” and the prediction accuracy “PCC” of our model with the top *k* features for all steps. The results show that, when *k* = 57, the PCC of our model reaches a maximum of 0.722. Thus, we choose *k* = 57 as the threshold of the feature selection algorithm.

**Figure 2:**
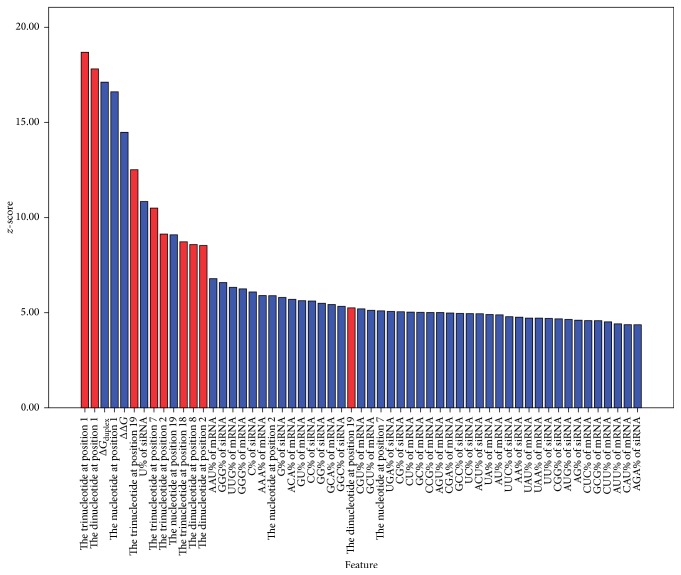
The 57 features selected by the BSFS method.

As shown in Figure 2, 57 features are selected by the BSFS algorithm and ranked in descending order according to *z*-score. The higher the *z*-score, the stronger the predictive ability of the feature. There are ten features proposed by our paper in the selective feature set, including the trinucleotides at positions 1, 2, 7, 18, and 19 and the dinucleotides at positions 1, 2, 8, and 19. Significantly, Takahashi noted the terminal bps of RNA (positions 19–21) provide Argonaute protein binding sites [26]. Our results show that “CUG” occurred most often at this position in potent siRNAs. The Argonaute protein is the endonuclease of RNA-induced silencing complexes (RISC) and cleaves the target mRNA whose sequence is complementary to the guide strand of siRNA [26]. We consider that, because the trinucleotide at position 19 is the binding site of the Argonaute protein, it will influence siRNA activity. However, further experiments are needed to validate if the Argonaute protein prefers to bind to potent siRNAs with specific trinucleotides at position 19.

**Figure 3:**
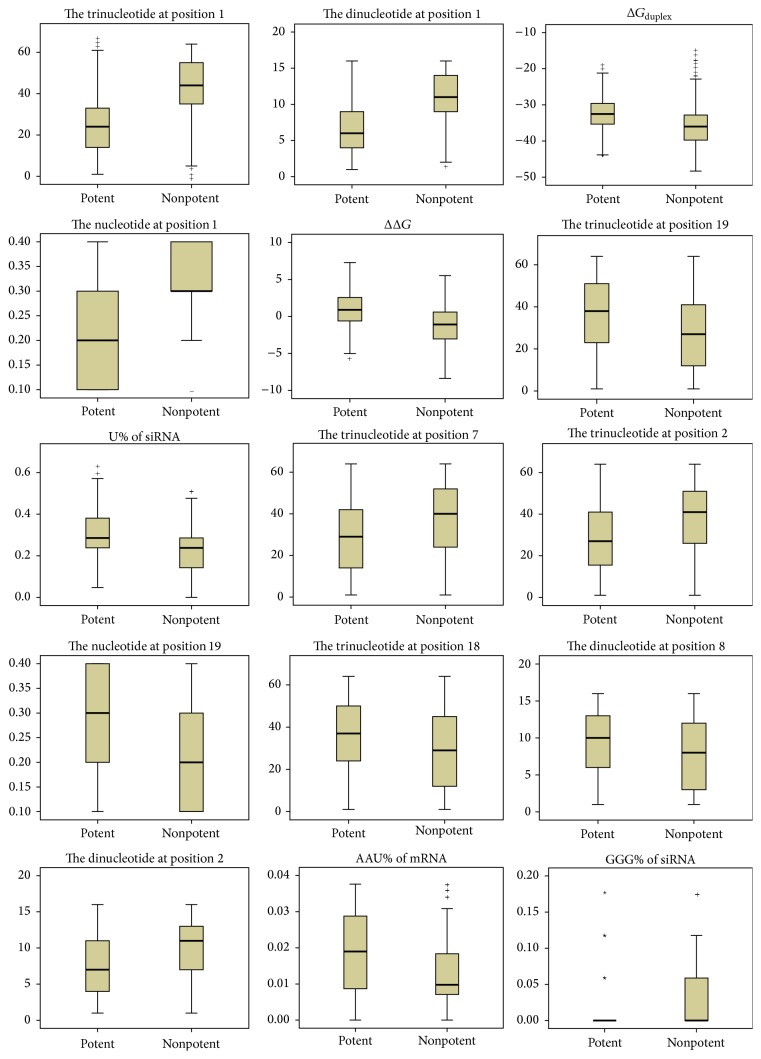
Boxplots of the top 15 features. For each plot, the left side represents potent siRNAs, and the right side represents nonpotent siRNAs.

Some other features previously proven to be associated with silencing efficacy are selected, including the nucleotides at positions 1, 2, 7 and 19; thermodynamic stability Δ*G*_duplex_ and ΔΔ*G*; and U%, GGG%, C%, G%, CC%, GG%, GGC%, UGA%, CG%, GCC%, UC%, ACU%, UUC%, AA%, UU%, CGG%, AUG%, AG%, and AGA% of siRNA; AAU%, UUG%, GGG%, AAA%, ACA%, GU%, GCA%, CGU%, GCU%, CU%, GC%, CCG%, AGU%, CGA%, UA%, AU%, UAU%, UAA%, CUC%, GCG%, CUU%, AUU%, and CAU% of mRNA. Graphical boxplots are shown in Figure 3 to display the spread of potent and nonpotent siRNAs for the top 15 features.

### 3.3. Comparison of Algorithms

After finding the optimal feature set, the final model, siRNApred, was created. The parameters *N* and *Mtry* are the number of decision trees to be grown in the forest and the number of variables to split at each node, respectively. The default *N* and *Mtry* are 500 and *D*/3. *D* is the number of features. To find the optimal parameters, we used a grid search method with the step size of 100 and 1. The final results are *N* = 1000 and *Mtry* = 24. The PCC between the observed and predicted siRNA activities of our model with these parameters is 0.722, which is a 1.7% improvement compared to the model with default parameters. However, the results are not sensitive to *Mtry* over the range 24–30 according to our experimental results.

To test the performance of siRNApred, we compared our model with the most state-of-the-art methods for siRNA activity prediction recently reported in the literature. Two experiments were carried out in the same conditions and the comparative evaluation is as follows.

First, our method was compared with Biopredsi [20], *i*-score [23], ThermoComposition-21 [21], and DSIR [22]. All the algorithms were trained using Huesken_train and tested on Huesken_test.  Table 4 shows that the PCC between observed and predicted siRNA activities of our model tested on Huesken_test is 0.722, which is 9.39%, 10.39%, 9.56%, and 7.76% higher than the other four algorithms.

**Figure 4:**
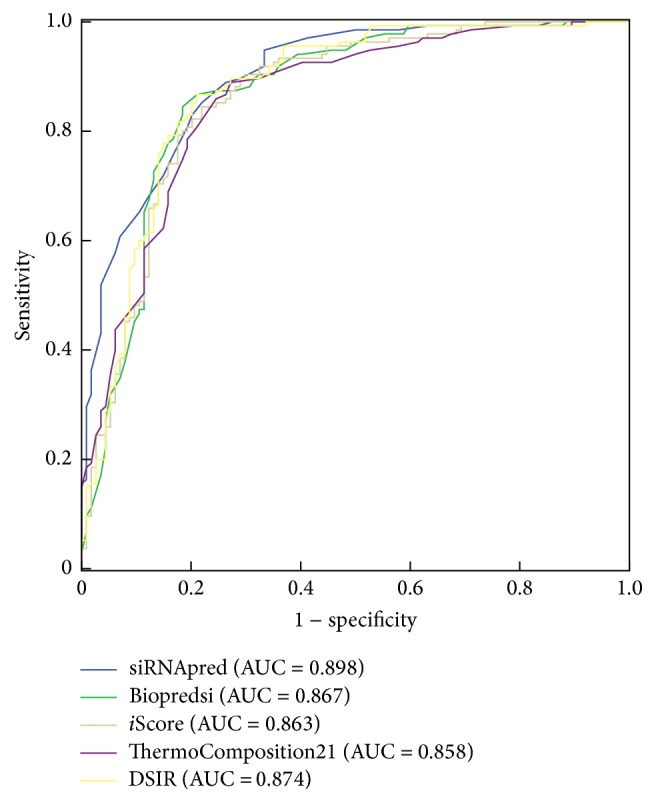
ROC curves of the five algorithms.

In addition, the ROC curves combining both sensitivity and specificity of the five methods are plotted (Figure 4). For ROC analysis, siRNAs that produce at least 70% target gene knockdown were accepted as active siRNAs, and those below 70% were considered inactive siRNA. We calculated an AUC of 0.898 for our model, which is better than those obtained from Biopredsi, *i*-score, ThermoComposition-21, and DSIR.

In siRNA design, more inactive siRNAs predicted as active siRNAs will increase the experimental cost, so siRNA design tools are expected to be capable of rejecting as many false positives as possible and retain the maximum number of true positives. Consequently, we should focus on the area that has higher specificity and compare the sensitivities among different algorithms in this area. Figure 4 shows that in the higher specificity area, siRNApred outperforms all other algorithms.  Table 5 shows two group sensitivities of all the algorithms. When the specificity of all algorithms is 96.5%, the sensitivity of our method is 51.9%. The value is higher than Biopredsi, *i*-score, ThermoComposition-21, and DSIR, which is 16.3%, 24.4%, 28.9%, and 20%, respectively. Our model also performs best when the specificity of all the algorithms is 99.1%. The results demonstrate that our method had more advantages than the other four algorithms for siRNA design.

A second experiment was conducted to compare our model with the other nine models, including the first-generation siRNA design algorithms Reynolds [11], Ui-Tei [14], Amarzguioui [12], Katoh [9], Hsieh [33], and Takasaki [34] and the second-generation algorithms Biopredsi, *i*-score, ThermoComposition-21, and DSIR. All the algorithms were trained on Huesken_train and tested on the three independent datasets of Vickers, Reynolds, and Harborth.

**Figure 5:**
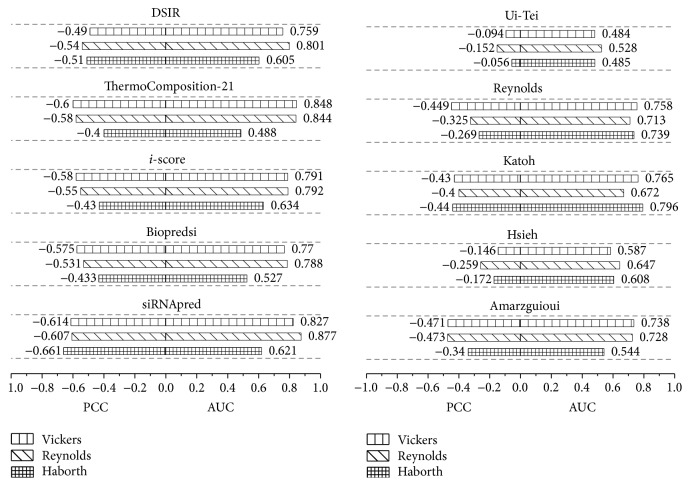
Comparisons of ten algorithms using the three independent datasets of Vickers, Reynolds, and Harborth.

Figure 5 shows that siRNApred achieves the highest PCC compared to all nine models on all three independent testing datasets and obtained a higher AUC except when tested on Vickers' dataset. Otherwise, siRNApred produces more stable results across each of the independent siRNA datasets. In addition, the results show that both the PCC and AUC of the first-generation siRNA design algorithms are lower than the second-generation algorithms.

It was found that siRNApred is more stable and effective than other models in the two experiments. The reason may be that our model takes account into the influence of di- and trinucleotides and removes several redundant features. The comparison results demonstrated that prediction accuracy can be improved significantly when considering the 2-3NTs of siRNA guide strand.

## 4. Conclusions

Activity prediction of siRNA is a critical step for the successful implementation of RNAi. In this study, we introduced 2-3NTs as our new features. A new mixed 230-dimensional feature set was formed by combining 191 traditional features and our 39 proposed features. Since there were many potential features, the BSFS method based on RF-variable importance was proposed to select the optimal feature set. A total of 57 features were selected as input vectors of the RF model to predict siRNA activity, and nine of our proposed features were included. Significantly, the trinucleotide motif at position 19 was included in the selected feature set, which is the binding site of the Argonaute protein. We found that “CUG” occurred most often at position 19 of potent siRNAs. Further experiments are needed to validate if the Argonaute protein prefers to bind to potent siRNAs possessing a specific trinucleotide at position 19. Finally, we describe a highly accurate and reliable tool called “siRNApred.” It can design effective siRNAs for an input mRNA using an optimal feature set. The experimental comparative evaluation on commonly used datasets showed that siRNApred produced better results than first-generation and second-generation siRNA design methods. Consequently, we consider siRNApred a worthy tool for efficient siRNA design.

## Figures and Tables

**Algorithm 1:**
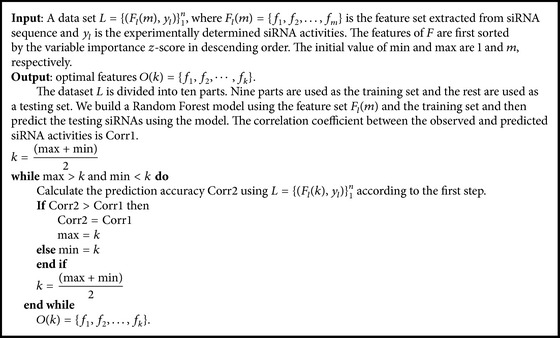
The calculation process of threshold *k*.

**Table 1:** Primary dinucleotides with minimal *p* value.

Position	Dinucleotide motif	Freq (*P*)	Freq (*N*)	Type of corr.	*p* value
1	UU1	178/1218	25/1213	Positive	9.45*e* − 30
	GG1	36/1218	159/1213	Negative	1.52*e* − 20
2	UA2	73/1218	32/1213	Positive	4.62*e* − 5
	GC2	48/1218	96/1213	Negative	3.26*e* − 5
3	AA3	76/1218	53/1213	Positive	0.0397
	CC3	57/1218	91/1213	Negative	0.0036
4	UU4	111/1218	69/1213	Positive	0.0013
	CC4	60/1218	107/1213	Negative	0.0001
5	AU5	94/1218	56 /1213	Positive	0.0015
	CC5	66/1218	102/1213	Negative	0.0036
6	UU6	117/1218	63/1213	Positive	3.19*e* − 5
	CC6	47/1218	110/1213	Negative	1.63*e* − 7
7	UU7	104/1218	67/1213	Positive	0.0036
	CA7	70/1218	120/1213	Negative	0.0001
8	CG8	32/1218	51/1213	Negative	0.0323
9	CA9	108/1218	66/1213	Positive	0.0010
	GU9	56/1218	84/1213	Negative	0.0138
10	AU10	101/1218	62/1213	Positive	0.0017
	CC10	63/1218	96/1213	Negative	0.0062
11	AA11	74/1218	46/1213	Positive	0.0094
	GG11	78/1218	111/1213	Negative	0.0114
12	CG12	32/1218	56/1213	Negative	0.0086
13	AU13	108/1218	65/1213	Positive	0.0008
	GG13	59/1218	114/1213	Negative	1.22*e* − 5
14	UU14	105/1218	72/1213	Positive	0.0108
	GG14	60/1218	110/1213	Negative	6.10*e* − 5
15	CA15	113/1218	74/1213	Positive	0.0033
	GG15	72/1218	108/1218	Negative	0.0048
16	AC16	82/1218	46/1213	Positive	0.0012
	GG16	68/1218	137/1213	Negative	3.82*e* − 7
17	AC17	80/1218	45/1213	Positive	0.0014
	GA17	51/1218	95/1213	Negative	0.0002
18	UC18	114/1218	69/1213	Positive	0.0006
	AA18	29/1218	87/1213	Negative	2.76*e* − 8
19	CU19	124/1218	53/1213	Positive	3.23*e* − 8
	AC19	30/1218	63/1213	Negative	0.0004
20	UG20	146/1218	67/1213	Positive	1.59*e* − 8
	CC20	52/1218	101/1213	Negative	3.73*e* − 5

**Table 2:** Primary trinucleotides with minimal *p* value.

Position	Trinucleotide motif	Freq (*P*)	Freq (*N*)	Type of corr.	*p* value
1	UUG1	52/1218	5/1213	Positive	9.48*E* − 10
	GGG1	4/1218	50/1213	Negative	1.90*E* − 10
2	UUA2	14/1218	4/1213	Positive	0.0184
	GCC2	10/1218	33/1213	Negative	0.0004
3	AUU3	28/1218	9/1213	Positive	0.0009
	CAC3	9/1218	29/1213	Negative	0.0005
4	UAU4	19/1218	5/1213	Positive	0.0021
	CCA4	19/1218	41/1213	Negative	0.0019
5	AUU5	29/1218	11 /1213	Positive	0.0021
	CCC5	6/1218	30/1213	Negative	2.59*E* − 05
6	UUU6	40/1218	12/1213	Positive	4.53*E* − 05
	CCA6	10/1218	41/1213	Negative	5.20*E* − 06
7	UCU7	37/1218	18/1213	Positive	0.005
	CGU7	3/1218	16/1213	Negative	0.0013
8	ACA8	29/1218	13/1213	Positive	0.0066
	AAU8	8/1218	28/1213	Negative	0.0004
9	CAA9	26/1218	7/1213	Positive	0.0004
	AUU9	12/1218	30/1213	Negative	0.0024
10	ACA10	35/1218	11/1213	Positive	0.0002
	CGA10	2/1218	12/1213	Negative	0.0036
11	CUA11	32/1218	13/1213	Positive	0.0022
	GCG11	6/1218	23/1213	Negative	0.0007
12	AUU12	30/1218	11/1213	Positive	0.0014
	GGG12	9/1218	31/1213	Negative	0.0002
13	UUU13	33/1218	16/1213	Positive	0.0074
	CCG13	6/1218	20/1213	Negative	0.0028
14	CCA14	36/1218	16/1213	Positive	0.0026
	CCC14	6/1218	21/1213	Negative	0.0018
15	UAU15	16/1218	4/1213	Positive	0.0036
	UGG15	19/1218	46/1218	Negative	0.0003
16	ACU16	31/1218	12/1213	Positive	0.0018
	CGA16	1/1218	10/1213	Negative	0.0032
17	CUG17	49/1218	21/1213	Positive	0.0004
	GUU17	9/1218	34/1213	Negative	5.57*E* − 05
18	UCU18	43/1218	11/1213	Positive	5.54*E* − 06
	AAA18	8/1218	28/1213	Negative	0.0004
19	CUG19	61/1218	16/1213	Positive	9.70*E* − 08
	AGA19	7/1218	31/1213	Negative	4.05*E* − 05

**Table 3:** The performance of our model with the top *k* features.

	Number of features (*k*)	Pearson Correlation Coefficient (PCC)
1	230	0.705
2	230/2 = 115	0.713
*3*	*115/2* =* 57*	*0.722*
4	57/2 = 28	0.712
5	28 + (57 − 28)/2 = 42	0.720
6	42 + (57 − 42)/2 = 49	0.721
7	49 + (57 − 49)/2 = 53	0.721
8	53 + (57 − 53)/2 = 55	0.719
9	55 + (57 − 55)/2 = 56	0.721

**Table 4:** PCC between observed and predicted siRNA activities for five algorithms.

Method	PCC (*r*)
Biopredsi	0.660
*i*-score	0.654
ThermoComposition-21	0.659
DSIR	0.670
*siRNApred*	*0.722*

**Table 5:** The five algorithms' sensitivities in the high specificity area.

Method	Sensitivity (96.5% specificity)	Sensitivity (99.1% specificity)
*siRNApred*	*51.9%*	*29.6%*
Biopredsi	16.3%	8.1%
*i*-score	24.4%	6.7%
ThermoComposition-21	28.9%	18.5%
DSIR	20.0%	10.4%

## References

[1] Timmons L., Fire A. (1998). Specific interference by ingested dsRNA. *Nature*.

[2] Montgomery M. K., Xu S., Fire A. (1998). RNA as a target of double-stranded RNA-mediated genetic interference in Caenorhabditis elegans. *Proceedings of the National Academy of Sciences of the United States of America*.

[3] Elbashir S. M., Harborth J., Lendeckel W., Yalcin A., Weber K., Tuschl T. (2001). Duplexes of 21-nucleotide RNAs mediate RNA interference in cultured mammalian cells. *Nature*.

[4] Novina C. D., Sharp P. A. (2004). The RNAi revolution. *Nature*.

[5] Aagaard L., Rossi J. J. (2007). RNAi therapeutics: principles, prospects and challenges. *Advanced Drug Delivery Reviews*.

[6] McMillen C. M., Beezhold D. H., Blachere F. M., Othumpangat S., Kashon M. L., Noti J. D. (2016). Inhibition of influenza A virus matrix and nonstructural gene expression using RNA interference. *Virology*.

[7] Wang F., Sun Y., Ruan J. (2016). Using small RNA deep sequencing data to detect human viruses. *BioMed Research International*.

[8] Wang T., Shigdar S., Shamaileh H. A. (2017). Challenges and opportunities for siRNA-based cancer treatment. *Cancer Letters*.

[9] Katoh T., Suzuki T. (2007). Specific residues at every third position of siRNA shape its efficient RNAi activity. *Nucleic Acids Research*.

[10] Elbashir S. M., Harborth J., Weber K., Tuschl T. (2002). Analysis of gene function in somatic mammalian cells using small interfering RNAs. *Methods*.

[11] Reynolds A., Leake D., Boese Q., Scaringe S., Marshall W. S., Khvorova A. (2004). Rational siRNA design for RNA interference. *Nature Biotechnology*.

[12] Amarzguioui M., Prydz H. (2004). An algorithm for selection of functional siRNA sequences. *Biochemical and Biophysical Research Communications*.

[13] Khvorova A., Reynolds A., Jayasena S. D. (2003). Functional siRNAs and miRNAs exhibit strand bias. *Cell*.

[14] Ui-Tei K., Naito Y., Takahashi F. (2004). Guidelines for the selection of highly effective siRNA sequences for mammalian and chick RNA interference. *Nucleic Acids Research*.

[15] Schubert S., Grünweller A., Erdmann V. A., Kurreck J. (2005). Local RNA target structure influences siRNA efficacy: systematic analysis of intentionally designed binding regions. *Journal of Molecular Biology*.

[16] Luo K. Q., Chang D. C. (2004). The gene-silencing efficiency of siRNA is strongly dependent on the local structure of mRNA at the targeted region. *Biochemical and Biophysical Research Communications*.

[17] Yiu S. M., Wong P. W. H., Lam T. W. (2005). Filtering of ineffective siRNAs and improved siRNA design tool. *Bioinformatics*.

[18] Ren Y., Gong W., Xu Q. (2006). siRecords: an extensive database of mammalian siRNAs with efficacy ratings. *Bioinformatics*.

[19] Sætrom P., Snøve O. (2004). A comparison of siRNA efficacy predictors. *Biochemical and Biophysical Research Communications*.

[20] Huesken D., Lange J., Mickanin C. (2005). Design of a genome-wide siRNA library using an artificial neural network. *Nature Biotechnology*.

[21] Shabalina S. A., Spiridonov A. N., Ogurtsov A. Y. (2006). Computational models with thermodynamic and composition features improve siRNA design. *BMC Bioinformatics*.

[22] Vert J.-P., Foveau N., Lajaunie C., Vandenbrouck Y. (2006). An accurate and interpretable model for siRNA efficacy prediction. *BMC Bioinformatics*.

[23] Ichihara M., Murakumo Y., Masuda A. (2007). Thermodynamic instability of siRNA duplex is a prerequisite for dependable prediction of siRNA activities. *Nucleic Acids Research*.

[24] Matveeva O., Nechipurenko Y., Rossi L. (2007). Comparison of approaches for rational siRNA design leading to a new efficient and transparent method. *Nucleic Acids Research*.

[25] Thang B. N., Ho T. B., Kanda T. (2015). A semi-supervised tensor regression model for siRNA efficacy prediction. *BMC Bioinformatics*.

[26] Takahashi T., Zenno S., Ishibashi O., Takizawa T., Saigo K., Ui-Tei K. (2014). Interactions between the non-seed region of siRNA and RNA-binding RLC/RISC proteins, Ago and TRBP, in mammalian cells. *Nucleic Acids Research*.

[27] Harborth J., Elbashir S. M., Vandenburgh K. (2003). Sequence, chemical, and structural variation of small interfering RNAs and short hairpin RNAs and the effect on mammalian gene silencing. *Antisense and Nucleic Acid Drug Development*.

[28] Vickers T. A., Koo S., Bennett C. F., Crooke S. T., Dean N. M., Baker B. F. (2003). Efficient reduction of target RNAs by small interfering RNA and RNase H-dependent antisense agents. A comparative analysis. *Journal of Biological Chemistry*.

[29] Xia T., SantaLucia J., Burkard M. E. (1998). Thermodynamic parameters for an expanded nearest-neighbor model for formation of RNA duplexes with Watson-Crick base pairs. *Biochemistry*.

[30] Teramoto R., Aoki M., Kimura T., Kanaoka M. (2005). Prediction of siRNA functionality using generalized string kernel and support vector machine. *FEBS Letters*.

[31] Liu Y., Chang Y., Zhang C. (2013). Influence of mRNA features on siRNA interference efficacy. *Journal of Bioinformatics and Computational Biology*.

[32] Breiman L. (2001). Random forests. *Machine Learning*.

[33] Hsieh A. C., Bo R., Manola J. (2004). A library of siRNA duplexes targeting the phosphoinositide 3-kinase pathway: determinants of gene silencing for use in cell-based screens. *Nucleic Acids Research*.

[34] Takasaki S., Kotani S., Konagaya A. (2004). An effective method for selecting siRNA target sequences in mammalian cells. *Cell Cycle*.

